# How Does Working Memory Enable Number-Induced Spatial Biases?

**DOI:** 10.3389/fpsyg.2016.00977

**Published:** 2016-06-29

**Authors:** Elger Abrahamse, Jean-Philippe van Dijck, Wim Fias

**Affiliations:** Department of Experimental Psychology, University of GhentGhent, Belgium

**Keywords:** numerical cognition, number-space associations, SNARC effect, working memory, serial order

## Abstract

Number-space associations are a robust observation, but their underlying mechanisms remain debated. Two major accounts have been identified. First, spatial codes may constitute an intrinsic part of number representations stored in the brain – a perspective most commonly referred to as the Mental Number Line account. Second, spatial codes may be generated at the level of working memory when number (or other) representations are coordinated in function of a specific task. The aim of the current paper is twofold. First, whereas a pure Mental Number Line account cannot capture the complexity of observations reported in the literature, we here explore if and how a *pure* working memory account can suffice. Second, we make explicit (more than in our earlier work) the potential building blocks of such a working memory account, thereby providing clear and concrete foci for empirical efforts to test the feasibility of the account.

In the human brain the processing of numbers gives rise to systematic spatial biases. Traditionally, these biases are explained by assuming long-term associations between individual numbers and a spatial code, such that these spatial codes are an intrinsic part of number representations in the brain (e.g., Hubbard et al., 2005). In a more recent alternative, van Dijck and Fias (2011), van Dijck et al. (2014), and Fias and van Dijck (2016) proposed the working memory account on number-space associations, in which numbers are linked to spatial codes in function of their ordinal position within a instructed sequence. Whereas ample empirical support exists to refute a pure Mental Number Line account, in the current paper we explore to what extent a pure working memory account can capture the apparent complexity and variety of findings in this domain.

Below we briefly sketch the classic long-term memory account and its basic pillars of support. Next, we work toward the presentation of an alternative account in which number-induced spatial biases are related to working memory. To this purpose, we first describe a set of the most critical empirical observations that has been argued to rule out a pure Mental Number Line account (see **Table 1** below, cases 3–9), as they point to a considerable flexibility in the mapping of numbers on space. Second, we outline the working memory account and make explicit – more than in our previous work – the precise mechanisms underlying this account. The latter is important both to capture the set of empirical observations that are at stake here, and to enable the account to become more readily available for focused empirical testing.

**Table 1:** Nine critical cases from the domain of number-space associations that are to be captured by a comprehensive working memory account.

Case	Observation	Working memory account	Key reference(s)
1	Smaller and larger number magnitudes facilitate left and right hand responding, respectively (SNARC effect).	Number items prompt canonical number set; targets are referentially coded with reference to the item midway; because the number set is operated on in a spatial format, spatial codes are generated relative the reference frame.	Dehaene et al., 1993

2	Smaller and larger number magnitudes facilitate left and right Posner dot detection, respectively (attentional SNARC effect).	Idem as above.	Fischer et al., 2003

3	The number ‘5’ primes left or right according to its use in overall ranges 5–9 or 1–5 (range effect).	Initially the number items prompt the canonical number set (1–9), but after some trials this item set is ‘pruned’ to match the actually used items in the experiment. Referential coding within the pruned set produces the range effect.	Dehaene et al., 1993; Fias et al., 1996

4	SNARC effect (as measured with parity judgment or magnitude comparison) disappears under a proper working memory load.	Working memory when loaded hinders the efficient maintenance of larger item sets.	Herrera et al., 2008; van Dijck et al., 2009

5	Performing a parity judgment task under instructed sequence load produces a SNARC effect with ascending and random sequences, but not with descending sequences.	Number items prompt the canonical number set (1–9), but descending instructed sequences hinder this process because they interfere strongly with the ascending order of the canonical number set.	Lindemann et al., 2008

6	Asking participants to imagine numbers on a clock-face induces number-specific attentional shifts corresponding to the position of the number on the clock in reference to the vertical clock midline.	Just as number items spontaneously prime a specific (left-to-right) orientation within working memory, task instructions can prime other spatial templates to become dominant.	Ristic et al., 2006

7	Earlier and later items from an arbitrary instructed sequence facilitate left and right responding, respectively (ordinal position effect).	A task-relevant, novel instructed sequence typically dominates the canonical number set in terms of activation in working memory, and referential coding within the spatially laid out, novel working memory set determines the generation of spatial codes.	van Dijck and Fias, 2011

8	Additive spatial biases from ordinal position and number magnitude processing.	Under some conditions, both the canonical number set and a novel instructed sequence may be activated simultaneously, such that referential coding occurs within both sets and thus generates independent spatial codes.	Ginsburg and Gevers, 2015; Huber et al., 2016

9	In an ordinal position judgment task (i.e., is a letter before or after a reference letter from the alphabet?), bilingual Hebrew-English participants showed both a left-right SNARC(-like) effect for English letters and a right-left SNARC(-like) effect for Hebrew letters.	English letters trigger a left-to-right orientation based on experience, and the reversed is true for Hebrew letters.	Shaki and Gevers, 2011

Overall, we defend the provocative but testable position that the working memory account suffices to understand number-space associations, thus refuting a dual processes model in which spatial codes derive both directly from long-term number representations and (indirectly) from processing numbers at the level of working memory (e.g., Ginsburg and Gevers, 2015; Huber et al., 2016).

## The Mental Number Line Account

Many researchers believe that humans represent numbers along the so-called Mental Number Line, a left-to-right oriented long-term memory representation on which numbers are organized from left to right based on their magnitude (i.e., canonical order).^1^ A core feature of this account is that spatial codes are intrinsically involved in number representations in the brain. Three major sources of evidence for the Mental Number Line may be identified. First, a major effect in the domain of numerical cognition is termed the Spatial Numerical Association of Response Codes (SNARC) effect (Dehaene et al., 1993). It involves the observation that participants respond faster to small numbers with left responses, and faster to large numbers with right responses. The SNARC effect is highly robust and was one of the main inspirations to the popularization of the Mental Number Line construct (Dehaene et al., 1993; Fias and Fischer, 2005; Hubbard et al., 2005; Gevers and Lammertyn, 2005).

Second, Fischer et al. (2003, p. 555) observed that number processing can directly bias spatial attention (i.e., attentional SNARC effect). Specifically, in an adapted version of the classical Posner cuing task it was shown that “merely looking at numbers causes a shift in covert attention to the left or right side, depending upon the number’s magnitude.” As detection targets occurred randomly on the left or right side of the screen – precluding successful prediction of the upcoming target location – the authors took this finding to reflect a rather obligatory activation of spatial representations as part of a number’s meaning.

Third, Zorzi et al. (2002) observed that right brain damaged patients suffering from hemispatial neglect, misjudge the midpoint of a numerical interval when asked to bisect it. For instance, in these patients 7 (instead of 5) is typically indicated as the midpoint of the interval between 1 and 9. Interestingly, this pattern of error in misplacement closely resembles that found for physical line bisection. That is, right brain damaged neglect patients typically misplace the subjective midpoint of a physical line toward the right, as if they neglect the left part of the line and perform the bisection on only the attended part (Marshall and Halligan, 1989). This study is thus taken to support the assumption that the Mental Number Line is isomorphically identical to physical lines – sharing a same metric – and thus going beyond the stage of mere metaphor (Umiltà et al., 2009).

## Observations-To-Be-Explained

Below we will outline various observations that challenge the strong notion of a Mental Number Line. Together with the regular SNARC and attentional SNARC effects (cases 1 and 2 from **Table 1**), these are important observations that need to be captured by any comprehensive account on number-space associations (see cases 3–9 of **Table 1**) – which the working memory account to-be-addressed below aspires to be.

With respect to the SNARC effect, it is well-known that the number ‘5’ relates to space in reference to the item set being used. That is, this number is faster responded to with a right response when the experiments contains the numbers ranging from 1 to 5, but is preferentially associated with a left response when the item set contains the numbers from 5 to 9 (Dehaene et al., 1993; Fias et al., 1996). This so-called *range effect* was one of the first types of ‘flexibility’ that would not be predicted from the strict notion of a Mental Number Line.

Other types of flexibility that oppose a pure Mental Number Line account soon followed. For example, the SNARC effect (as measured with parity judgment or magnitude comparison) disappears under a proper working memory load that sufficiently occupies the available working memory resources (Herrera et al., 2008; van Dijck et al., 2009; Ginsburg et al., 2014). The impact of working memory load may be modulated by other factors. For example, Lindemann et al. (2008) had participants perform a number parity task while maintaining in memory 3-item sequences that later needed to be recalled. Please note that, unlike the study by van Dijck and Fias (2011) that will be discussed below, the memory sequences in the study by Lindemann et al. (2008) were unrelated to the parity task. A typical SNARC effect was observed in maintenance conditions with memory sequences in ascending (e.g., 3-4-5) or arbitrary order (e.g., 5-3-4), while no SNARC effect was obtained when maintaining sequences in descending order (e.g., 5-4-3).

With respect to the attentional SNARC – the standard version of which does not always seem so robust (e.g., van Dijck et al., 2014; Zanolie and Pecher, 2014; Fattorini et al., 2015), and various studies suggest that – unlike what would be predicted by a pure Mental Number Line account – the effect depends on the mental set that is instructed to and/or adopted by the participants. For example, asking participants to imagine numbers on a clock-face induces number-specific attentional shifts corresponding to the position of the number on the clock in reference to the vertical clock midline (Ristic et al., 2006). Moreover, asking participants to shift attention toward the left (right) after seeing a large (small) number reverses the attentional SNARC effect (Galfano et al., 2006).

The most extreme case of ‘relaying’ the regular SNARC and attentional SNARC effects may be the work by van Dijck and colleagues. In recent years, they have collected substantial empirical support for the notion that ordinal position in working memory produces spatial biases. In their experiments, an arbitrary series of numbers (e.g., 3-1-8-6-4; hereafter referred to as the ‘instructed sequence’) is presented to participants on the center of the screen, and participants are asked to maintain the sequence in working memory such that they can later reproduce it. During the retention interval, participants perform a task on number items (for example, a magnitude or parity judgment task, or a Posner detection task) in which responses are required only when the current number is a member of the sequence in memory – such that working memory retrieval is needed. It is typically observed that the ordinal position of the number in the instructed sequence induces spatial bias. Specifically, numbers early within the instructed sequence are relatively associated with left, while later numbers in the instructed sequence are increasingly associated with right. This interaction between serial order in working memory and spatial processing has been replicated across numerous studies both at the level of response selection (van Dijck and Fias, 2011; Ginsburg et al., 2014; Ginsburg and Gevers, 2015; van Dijck et al., 2015a) and attentional orienting/selection (e.g., van Dijck et al., 2013, 2014; De Belder et al., 2015), and has been demonstrated to occur in a similar way with non-numerical items (e.g., letters, pictures of objects) that need to be categorized according to a specific classification rule (e.g., van Dijck and Fias, 2011; van Dijck et al., 2014). Importantly, De Belder et al. (2015) demonstrated an impact between space and ordinal position in working memory in the reversed direction, as exogenous lateral cues could facilitate retrieval from working memory when left and right cues were combined with retrieval of early and late items of the instructed sequence, respectively. Moreover, Rinaldi et al. (2015) showed how retrieval of items from working memory has an impact on spontaneous eye movements according to ordinal position within the instructed sequence.

In a recent study, Ginsburg and Gevers (2015) adjusted the work by van Dijck and colleagues discussed above in the sense that a so-called inducer phase was included in which participants were asked to respond to all numbers (i.e., no go/no-go instruction). Afterward, participants continued with the typical instructed sequence phase (i.e., go/no-go) as in, for example, van Dijck and Fias (2011). Across two experiments the authors observed additive effects on space of number magnitude and the number’s ordinal position within the instructed sequence. Indeed, Huber et al. (2016) even showed the co-existence of these effects *without* the additional respond-to-all manipulation. Huber et al. (2016) had participants perform a parity task on number items that were part of an instructed sequence, while items from outside the memorized sequence required withholding of responses (i.e., go/no-go; cf. van Dijck and Fias, 2011). Both the number of items in the memorized sequence and the overall number set employed (i.e., 1-9 versus 1-10) were manipulated. Huber et al. (2016) observed simultaneous impacts of both ordinal position within the instructed sequence and number-magnitude on spatial response selection. Notably, both Ginsburg and Gevers (2015) and Huber et al. (2016) concluded that these results refute a pure working memory account as number-magnitude still produces spatial codes – in line with the Mental Number Line account. They took this to propose a dual processes model in which spatial codes both derive directly from long-term memory and are formed at the level of working memory. Below we discuss how an extended working memory account captures their effects without moving toward a dual processes model.

Finally, Shaki and Gevers (2011) explored bilingual Hebrew-English participants performing an ordinal position judgment task (i.e., is a letter before or after a certain reference letter from the alphabet?). In their Experiment 1 they showed both a left-right SNARC(-like) effect for English letters and a right-left SNARC(-like) effect for Hebrew letters. Moreover, in their Experiment 2 they exploited the fact that Hebrew letters can flexibly be used to represent magnitude information (i.e., the “Gematria” system), and showed that when magnitude was emphasized in the instructions, Hebrew letters now showed a left–right SNARC effect.

Inspired by some of the observations above, a working memory account on number-space interactions was previously formulated to capture the apparent flexibility in number-space associations (Fias et al., 2011; Fias and van Dijck, 2016). Here we extend the previous formulations and make explicit its precise underlying mechanisms. The aim is to provide a single account that captures the entire set of empirical studies just outlined above, and which is explicit in its mechanisms such that it provides a clear starting point for future testing.

## Working Memory Account

Our working memory account on number-space associations builds on the increasingly popular notion that working memory emerges from active long-term memory representations (Cowan, 1999; Postle, 2006; Oberauer, 2009). In brief, we postulate that people build an experience-based mental ‘work space’ when dealing with verbal content like numbers in order to enable the processing of and operations on this content. This work space consists of a binding of the most relevant set(s) of verbal items to a specific spatial template that is selected from long-term memory on the basis of experience, and which is ‘filled in’ according to the most dominant experience with such content. The spatialized, verbal content in the work space generates spatial codes based on referential processes through which each upcoming target item is matched to the content in the work space. In what follows we will first elaborate on these building blocks of our working memory account in a relatively abstract fashion, and subsequently put them into context by discussing how the working memory account captures the empirical phenomena discussed above (i.e., **Table 1**). We will end with a brief discussion on why we do not defend a dual processes model in which number-specific spatial codes are present at the level of long-term memory (cf. Mental Number Line).

### The Basics Of The Working Memory Account

The five core assumptions of our working memory account are summarized in **Table 2**. Please note that whereas assumptions one to four were (implicit) part of the original working memory account as formulated by Fias et al. (2011), van Dijck and Fias (2011), van Dijck et al. (2014), and Fias and van Dijck (2016), the fifth assumption that we here propose is an extension that we believe helps to accommodate the findings by Lindemann et al. (2008), Ginsburg and Gevers (2015), and Huber et al. (2016). This is all elaborated on below.

**Table 2:** Five core assumptions underlying an extended working memory account on number-induced spatial biases.

Core assumptions	
1	Multiple number items are spatially coordinated at the level of working memory by binding them to an active spatial template from long-term memory
2	Number items trigger global left-to-right orientation in working memory due to experience
3	Long-term memory contains – besides item representations – a representation for ordered item sets that are used frequently and systematically (i.e., canonical number set 1–9)
4	Spatial codes are generated through referential coding of a current target’s match in the working memory set relative to the item midway the set
5	Multiple item sets can be active in working memory simultaneously

First, whenever a cognitive agent performs a task on items from a specific item set (such as during a typical experiment), the relevant items become active (i.e., working memory). To coordinate the active items, each item is bound to a specific coordinate within an existing, well-coordinated template from long-term memory (cf. ‘context templates’ as in position marking theories on serial order; e.g., Burgess and Hitch, 1999, 2006). We claim that these templates are spatial in nature, even for verbal items like numbers, letters, words, et cetera (e.g., Abrahamse et al., 2014; see also Oberauer, 2009). Hence, the agent forms what can be likened to a mental work space, with the purpose of facilitating the coordination of and the operation on the verbal items. Since the active item set is laid out in working memory along a spatial template, searching through and selecting from the working memory set is related to internal spatial attention (e.g., cf. Chun et al., 2011; van Dijck et al., 2013, 2014). The critical distinction between this ‘spatialization’ in working memory and the Mental Number Line is that in our account spatial codes derive from the task-specific and temporary binding of number items to a spatial template – whereas for the Mental Number Line account the spatial codes are intrinsic part of number representations in long-term memory.

Second, we assume that spatialization of numbers in working memory occurs within a horizontally outlined, left to right direction because of our experience with number items. We want to emphasize here that this mechanism is assumed to hold for any other type of verbal items (such as letters or words) that requires orderly arrangement; already early in our education number symbols are learned foremost as verbal items (possibly semantically grounded in sensory-motor systems that provide referents such as body, fingers, objects, et cetera; e.g., Fischer, 2012). The educational system mainly coordinates these items – at least in most European countries – from left to right within various 2-dimensional media such as paper or whiteboard (e.g., Chen and Verguts, 2010). We learn to read and write from left to right with verbal items such as numbers, letters, and words, and counting may also involve a left-to-right direction (e.g., Fischer, 2008; Lindemann et al., 2011). Broadly, via these experiences (that may involve numbers but are not exclusively restricted to these) from early life onward, verbal items become associated to left-to-right defined coordination. As a consequence, every time that we load working memory with verbal items such as numbers, the spatial template that is used in working memory to coordinate these items is ‘filled in’ from left-to-right. Please note that this differs from the notion of a Mental Number Line in the sense that number items are associated with a global spatial orientation, but not to specific spatial codes that are part of their semantic foundation.

Third, besides individual item representations *per se* and their link to a specific spatial direction, we assume a third long-term memory component. In our previous work we did not explicitly consider consolidation of item sets to long-term memory. Yet, when dealing so extensively with numbers during our lives, it will come as no surprise that the most common numbers and their relationships become more or less hard-wired in the brain. We argue that one such feature is the orderly arrangement of the numbers 1–9 – a set of which both the items and their serial order relations have consolidated into an overlearned, long-term memory representation due to systematic and frequent use (e.g., counting; the use of the numerical pad on the keyboard; the use of a mobile phone).^2^ We refer to this long-term memory representation – which may underlie, among others, the well-known numerical distance effect (e.g., Moyer and Landauer, 1967) – as the *canonical number set*, which may be primed (i.e., activated) every time that we are committed to processing number items between 1 and 9. Please note that in the working memory account we explicitly claim that only *order* and no *spatial* information needs to be co-represented at this long-term level – which is the main distinction between the canonical number set and the Mental Number Line (for elaboration see below the section “Why Not a Dual Processes Model?”).

Fourth, spatial codes are generated from referential coding processes that are at play when a currently presented item is matched to the active, spatially defined ‘set template.’ For example, in an experiment using the numbers between 1 and 9, a currently presented target item “2” may be referenced to the item midway the active canonical number set – that is, the number “5” – and thus result in a relative left-ward code. Hence, each target on the screen is compared and matched to the spatially coordinated items in working memory (i.e., all active items), and a spatial code is generated based on the target’s ‘hit’ relative to the reference frame provided by the active number set (e.g., the item midway the set).^3^ The key implication is that it is the ordinal position within the specific ordering of items in working memory that determines the direction of the spatial codes – in reference to the overall active set. Since internal and external attentional selection procedures interface with each other, these referential coding processes in working memory bias external selection (e.g., attentional selection, response selection) that can be observed in behavioral performance measures. It is notable that referential coding is actually at the basis of the semantic system that provides meaning to numbers in the first place (e.g., ‘two’ only has meaning when referred to being more than ‘one’ and less than ‘three’). Moreover, referential coding is a key mechanism through which working memory produces spatial codes in other domains. The spatial stimulus–response compatibility effect – or Simon effect – for example, is largely attributed to the referencing of the laterally presented stimulus to the midpoint of one or more reference frames (e.g., Umiltà and Liotti, 1987; Hommel, 1993, 2011).

Fifth and last, we extend our working memory account as previously formulated with the assumption that multiple item sets can be active in working memory at any given time. In other domains such as task switching it has been shown that multiple mental (task) sets can be maintained and impact behavior concurrently – even when merely instructed. For example, task-rule congruency effects have been observed on the basis of instructed – but not yet applied – task sets (Liefooghe et al., 2012). Moreover, an item can be referenced within multiple spatial reference frames at the same time (e.g., Umiltà and Liotti, 1987). Building on these well-known observations, we assume that it is possible that each current target item can be spatially referenced within multiple active number sets in working memory at the same time, and bias attentional and/or response selection accordingly. Number sets can be formed *de novo* such as when asking participants to memorize an arbitrary sequence of numbers (i.e., instructed sequence; e.g., van Dijck et al., 2013), but number items (when sufficiently processed) also activate the canonical number set (1–9) – and under some conditions both become active in working memory together. Whereas the former is active whenever it is relevant to the task at hand (e.g., to determine whether a trial is go or no-go, or to achieve later recall; cf. van Dijck et al., 2013), the latter may be at play – among others – on the mere fact that it is part of the semantic system that provides meaning to numbers. Furthermore, the simultaneous activation of multiple item sets in working memory can be very efficient in a dual task situation where two (equally important) tasks are to be performed on a specific stimulus set (e.g., parity judgment and sequence recall).

Below we describe how this working memory account accommodates both the typical SNARC effects and its shown flexibility.

## Observations Explained

We now turn back to the list of critical observations discussed above (see **Table 1**), and outline how the working memory account sketched in the previous section can explain these observations.

The canonical number set may become active through the use of numbers as targets in an experiment – just because of the association between each number and the entire set. Hence, in a typical SNARC experiment, using numbers between 1 and 9 will activate the canonical number set (i.e., working memory), and this will prompt referential coding between any current target item and its relative position within the active canonical number set – thus generating spatial codes underlying the SNARC effect. Hence, (attentional) SNARC effects are actually ordinal position effects of working memory functioning instead of magnitude-based spatial biases from long-term memory (cf. van Dijck and Fias, 2011; van Dijck et al., 2014). Notably, whereas the SNARC effect itself is rather robust, the attentional SNARC is not (e.g., van Dijck et al., 2014; Zanolie and Pecher, 2014; Fattorini et al., 2015). This may be explained by the fact that – other than with the parity or magnitude task in the typical SNARC experiment – in an attentional SNARC experiment the numbers (which are task-irrelevant) are not necessarily processed deeply enough to activate the canonical number set.

Moreover, when loading working memory with a novel, instructed item sequence, each current target item will now be matched against this instructed set such that spatial biases through referential coding result in an effect of ordinal position within the instructed sequence on response selection and/or attentional orientation (van Dijck and Fias, 2011; van Dijck et al., 2013, 2014; Ginsburg et al., 2014; De Belder et al., 2015; Ginsburg and Gevers, 2015; Rinaldi et al., 2015).^4^ Indeed, under normal circumstances a cognitive agent will not take the effort of maintaining two item sets – the canonical number set and the instructed sequence – in working memory at the same time, and this explains why we do not typically see additive effects of canonical number set (i.e., SNARC effect) and instructed sequence (cf. van Dijck et al., 2013, 2014): maintenance of the novel item set (i.e., instructed sequence) discourages the activation of the canonical number set (1–9) in working memory. This may also explain why the SNARC effect sometimes disappears when loading working memory (e.g., Herrera et al., 2008; van Dijck et al., 2009).

The typical left–right direction of the SNARC effect has been shown to be easily overwritten toward alternative spatial lay-outs, and the working memory account can capture such flexibility. Take for example the study by Ristic et al. (2006). From the notion that an item set is laid out in working memory along a spatial template in order to facilitate processing of and operating on the items, it can be possible to prime the use of specific types of spatial template (just like the typical left–right orientation is primed by, for example, the use of number items): requesting participants to imagine numbers on a clock-face will induce a clock-based lay-out in working memory – and spatial biases according to referencing numbers according to the clock midline.

As another example of flexibility, the range effect follows directly from the fact that referencing ‘5’ within item sets involving the numbers either between 1 and 5, or between 5 and 9, results in different relative codes. From the perspective of the working memory account, what may happen here is that initially the number items prompt the canonical number set (1–9), but after some trials this item set is ‘pruned’ to match the actually used items in the experiment (i.e., the item set in working memory is reduced to accommodate either range 1–5 or range 5–9). Consequently, the ‘filling in’ of the spatial template in working memory is adjusted accordingly such that the item ‘5’ is either the most left (range 5–9) or right (range 1–5) item in working memory.

Let us now discuss three related studies (i.e., Lindemann et al., 2008; Ginsburg and Gevers, 2015; Huber et al., 2016) that were discussed above, and which recently were reported as challenges to a pure version of the working memory account in the sense that they could indicate dual processes from which spatial codes are generated (i.e., intrinsically spatial codes from long-term number representations, and spatialization at the level of working memory). We believe that the current, extended formulation of the working memory account can accommodate these challenges.

With respect to the findings by Ginsburg and Gevers (2015), we believe that when providing participants with an experimental manipulation that strongly activates the canonical number set representation, cognitive agents may be prompted to co-activate in working memory both the instructed sequence and canonical number set. As a result, each target item will be matched against two templates, such that referential coding within each template generates an independent spatial code. Huber et al. (2016) did not include a focused experimental manipulation that may have activated the canonical number set above and beyond what is the case for the standard design. It can of course always be the case that one population is more inclined to co-activate the canonical number set due to, for example, more extensive experience with mathematics or so, but this may be a dissatisfying explanation here. Yet, Huber et al. (2016) switched number ranges half-way through the experiment (e.g., from range 1–9 to range 1–10), and this may have drawn attention to the canonical number set more than is the case for a typical SNARC study. Overall, the point is that there is no reason to assume from these findings the existence of a Mental Number Line (in addition to spatialization at the level of working memory) because the working memory account can accommodate them – even though future work will be needed to precisely understand how.

To return to the study by Lindemann et al. (2008), we believe that this study touches upon the dynamics and boundary conditions of activating multiple item sets (e.g., an instructed sequence as well as the canonical number set) in working memory. To recap, they observed a SNARC effect only for ascending and arbitrary instructed sequences, and not for descending ones. We believe that, first, the three-item sequences used in their study probably did not load working memory as extensively as for example the study by van Dijck et al. (2009) – who used sequence length according to working memory span. This may have allowed the agent to keep active both the instructed sequence and the canonical number set, and matching a current target item to the latter may have resulted in SNARC effects. Yet, the descending instructed sequence condition may have especially interfered with the ascending order of the canonical number set, and this interference may have discouraged active maintenance of the canonical number set. Hence, interference between different item sets (instructed or long-term) may greatly determine the extent to which both are co-activated in any particular design – and thus which spatial biases will be observed.

Finally, above we described how Shaki and Gevers (2011) observed reversed spatial biases for English versus Hebrew letters in bilingual Hebrew-English participants. First, the fact that letters generate spatial biases would require defenders of the Mental Number Line construct to assume a more general spatial lay-out in long-term memory – for example in terms of magnitude coding (e.g., Walsh, 2003). Conversely, our working memory account was never about numbers *per se* in the first place – it holds for all types of information that can be ordinally arranged in working memory, such as numbers, words, and also letters. Yet, where does the reversal come from? We believe that the reversal fits the notion that letter items prompt a global spatial orientation that that they most strongly relate to, such that spatial biases are triggered accordingly in working memory. In this specific population, English letters trigger a left-to-right orientation based on experience, and the reversed is true for Hebrew letters. Moreover, in their Experiment 2 they exploited the fact that Hebrew letters can flexibly be used to represent magnitude information (i.e., the “Gematria” system), and showed that when magnitude was emphasized in the instructions, Hebrew letters now showed a left-right SNARC effect. Our working memory account can explain this through the notion that within the context of magnitude decisions, the dominant direction is left to right in this population – due to, for example, their experience with reading and writing the typical English number system. Indeed, this type of result would be more difficult to explain without the flexibility that working memory brings to number-space associations.

Overall, the extended working memory account that was formulated above (cf. **Table 2**) may well be able to capture the large complexity of findings from the literature on number-space associations (cf. **Table 1**).

## Why Not A Dual Processes Model?

Above we argue that the systematic use of number items in a specific order results in orderly arranged, long-term item set representations (cf. the canonical number set). Yet, if the subsequent use of the ordered item set is systematically linked to a global spatial lay-out in working memory, then why wouldn’t direct associations between an item and a spatial code be formed in long-term memory? Theoretically, we do not find this possibility problematic, and indeed perhaps systematic use in working memory of an ordered set of number items within a left-to-right directed orientation may ultimately evolve to something like a Mental Number Line (i.e., including a spatial component in long-term memory). Yet, a pure working memory account is more parsimonious than a dual processes model that includes both long-term number-space associations (cf. Mental Number Line) and spatialization in working memory, because the former captures the complexity of the empirical database (cf. **Table 1** and our discussion above) through a single type of process. Hence, for as long as there is no empirical need to move toward a dual processes model, the working memory account that we defend here should be prioritized.

Moreover, we believe that there actually exist empirical reasons against a dual processes model in the sense that there are various studies in which magnitude (in other words, the canonical order) does not generate any spatial bias even with clear indications that it is processed. In our studies using instructed sequences, for example, we do not typically see additive effects of (magnitude-based) SNARC on top of our ordinal position effects (e.g., van Dijck et al., 2013, 2014; Ginsburg et al., 2014), while this would be strongly predicted if number items were associated to space by a hard-wired, long-term memory code.

More importantly, we believe that Experiment 1 from the study by van Dijck et al. (2014) indicates that referential coding (within working memory) is required for the generation of spatial codes – just processing numbers does not suffice. Specifically, in this experiment, a typical attentional SNARC task was combined with the instruction that at random moments in the experiment the participant could be asked to name the number that occurred in the trial that they had just finished. This additional task encourages participants on each trial to maintain in working memory only the number they have just seen, with referencing to other numbers possibly interfering with this memory instruction. Interestingly, despite the magnitude of the number (cf. its position within the canonical number set) showing a main effect on performance (indicating that magnitude was processed; see also Experiment 1 of van Dijck and Fias, 2011 and Experiment 2 of van Dijck et al., 2014) and despite reasonable scores on the secondary naming task (indicating that numbers were maintained in working memory) there was no effect on spatial response selection. We interpret this as indicating an absence of referential coding due to a strict focus on the single, to-be-maintained item without placing the item within the larger context of the overall canonical number set.

Finally, even in a typical parity judgment SNARC task, the spatial coding of numbers can be easily modified by subtle spatial manipulations (which is difficult to explain within a Mental Number Line account). For example, Fischer et al. (2010), showed that the placement of numbers in a text (i.e., with large (small) numbers systematically positioned on the left (right) of the paper or vice versa) that was read just before the administration of the parity judgment task, modulated the strength of the SNARC effect. Other examples of this flexibility were provided already above.

Critically, as long as the working memory account can accommodate the observations from the literature – including the studies reporting on additive effects of number magnitude (cf. canonical number set) and ordinal position within an instructed sequence – it will always be more parsimonious than a dual processes model that postulates both spatialization in working memory and spatially defined long-term number representations. In terms of the working memory account the long-term component is mainly related to serial order information – no one will disagree that human agents possess a long-term representation of a set of ordered number items – and currently there may not be sufficient reason to also assume a long-term spatial component to number representations (cf. Ginsburg and Gevers, 2015; Huber et al., 2016).

## Final Remarks and Future Challenges

Above we have made explicit the core mechanisms underlying our extended working memory account on number-space associations, and showed how this account can capture a large and complex empirical database. We hope that by making explicit (more than in our previous work) these core mechanisms, we here provided clear foci for future research and for putting our account to the test. Before accepting a dual processes model in which both long-term number-space associations and spatialization at the level of working memory are at play, it is critical to test – and possibly falsify – the more parsimonious working memory account. Such falsification attempts may focus on manipulations affecting the core components, such as referential coding, the nature of the most active item set, the maintenance of multiple item sets, or the shifting of attention through space.

To wrap up, we would like to briefly discuss four immediate challenges to the working memory account that present themselves at this point. First, our extended working memory account prompts exploration into the complex dynamics underlying working memory content and the bias it generates in spatial attention and/or response selection. Take for example the study by Ginsburg et al. (2014). In their Experiment 3, participants needed to maintain in working memory a sequence of items as in the earlier work by van Dijck and colleagues (e.g., van Dijck and Fias, 2011) – but with the one difference that during the retention interval all items in the magnitude task required a response (i.e., only go trials). In this design, a regular SNARC effect was observed across all items – both from within and from outside the instructed sequence. Moreover, for the items within the instructed sequence there was no impact on spatial processing observed for ordinal position. On the one hand, we believe these results are rather straightforward: within the context of a magnitude task, working with the canonically ordered item set may have been much more beneficial for the task at hand than would be the case for actively sorting out items within the instructed sequence, both because the former is directly compatible with the magnitude instructions and because the latter does not contain all items to be processed. On the other hand, they challenge the domain to further our understanding of *set shifting* in this particular context. What mechanism enables selection from the active sets in working memory? What determines which of the multiple sets in working memory gets to impact spatial processing (cf. output gating; Chatham et al., 2014)? Here, much may be learned from research on related cognitive control functions in other, related domains (e.g., task switching, attentional set shifting, working memory updating).

Second, while we here postulate that multiple item sets can be actively maintained in working memory – thereby accounting for simultaneous observation of typical SNARC and ordinal position effects – future work needs to address the related but alternative possibility that active item sets differ between trials, resulting in an overall impact of both only at the experiment level. Both can explain the simultaneous effect – at group level – of canonical and novel number sets on spatial processing.^5^ Future studies may want to explore if these effects are sensitive to different manipulations, and or whether they impact performance at different moments within an ongoing trial. The latter may be addressed by replicating the study by Ginsburg and Gevers (2015) while using mouse responses (instead of key-presses) to reveal the temporal dynamics of spatial biases on action control.

Third, future work will need to address how our working memory account relates to general models of working memory. There is increasing consensus that working memory emerges from the active parts of long-term memory (e.g., Cowan, 1999; Postle, 2006; Oberauer, 2009), and this fits with the notion that number-space associations arise from active item sets. Moreover, there is an intuitive link to the working model by Oberauer (2009) in particular. In this model, Oberauer distinguished between active long-term memory, a subdivision referred to as the region of direct access which is at heightened state of activation and comprises the means of novel bindings, and a focus of attention that selects single elements from the region of direct access. Since novel instructed sequences require the temporary binding between elements (cf. serial order), we would argue that these are mainly related to the region of direct access. Conversely, ordered item sets from long-term memory (i.e., the canonical set) contains order information such that it may not require binding in the region of direct access – and possibly have enhanced potency to work at the background (i.e., as activated long-term memory). Indeed, Nee and Jonides (2011) assumed that region of direct access and focus of attention may even differentially contribute to the maintenance of a instructed sequence (e.g., later items may still be in the focus of attention, while earlier items are within the region of direct access). These theoretical possibilities may drive future research on their impact on spatial behavior.

Fourth, a major theoretical challenge to our working memory account relates to the set of studies that have suggested that number-space associations are biologically endowed universals that do not emerge through experiences with language and/or culture (e.g., Dehaene et al., 2008; de Hevia and Spelke, 2009, 2010; Drucker and Brannon, 2014). Indeed, some go as far as to suggest that even newborn domestic chicks share the basis of what has developed into humans’ Mental Number Line (Rugani et al., 2015). We believe that the challenge will be to understand how the two perspectives – phylogenetic versus cultural influences on the development of number-space associations – are related to each other (Shaki and Fischer, 2015). In this respect it may be important to note that our closest evolutionary relatives, chimpanzees, link items from an *acquired* sequence to space in a similar way as humans do (Adachi, 2014); whereas this study fits with the notion that these number-space associations are acquired throughout life, it also goes against the idea that reading and writing are the fundamental factors in shaping these associations (as we have claimed above). Perhaps other factors can shape these associations such as the well-known leftward bias in spatial processing (Jewell and McCourt, 2000; Della Sala et al., 2010). Such biases – or analogs to them – may be shared across species. For example, the absence of a corpus callosum in chicks enhances hemispheric differences in the processing of information, which may lead to left-biased exploration behavior (Vallortigara and Andrew, 1991; Andrew and Rogers, 2002) due to the for example right-hemispheric dominance in reacting to novelty (Vallortigara et al., 1996; Tommasi et al., 2000). This could be one of mechanisms that gave rise to the number-space associations observed in new born chicks (Núñez and Fias, 2015). Above we postulate that experience (e.g., reading, writing, counting) has shaped (the direction of) spatial coding in working memory, but it will be a challenge to broaden the context and understand the joint impact of phylogenetic and cultural influences.

As a final note, we here have adhered to the notion of a Mental Number Line as the core long-term memory construct that drives number-space interactions. However, we must also acknowledge that other long-term memory accounts of the SNARC effect exist that relate the effect to associations between magnitude concepts (e.g., small, large) and spatial concepts (e.g., left, right) (Gevers et al., 2006; Proctor and Cho, 2006; for an overview see van Dijck et al., 2015b). These accounts, too, start from the notion that long-term representations underlie number-space interactions, and we believe that our extended working memory account may provide inspiration in terms of underlying mechanism also for these accounts.

## Author Contributions

All authors listed, have made substantial, direct and intellectual contribution to the work, and approved it for publication.

## Conflict of Interest Statement

The authors declare that the research was conducted in the absence of any commercial or financial relationships that could be construed as a potential conflict of interest.

## Footnotes

1 In fact, the direction of this organization (i.e., left-to-right versus right-to-left) is culturally determined and seems to strongly follow reading and writing direction (e.g., Göbel et al., 2011). Here we focus on Western cultures, which typically show a left-to-right orientation. See also below in the section “Observations Explained.”

2 When using items between 1 and 10, the same long-term set representation of 1–9 maybe activated but slightly adapted to also contain the number 10. In fact, such “long-term working memory” has been argued to be at the basis of expertise (e.g., Guida et al., 2012).

3 Here the precise scaling of space may be relevant in determining the ‘middle’ – the reference point – of a spatialized sequence in working memory (cf. Siegler and Opfer, 2003).

4 Effects on spatial behavior of random number generation (e.g., eye movements in Loetscher et al., 2010; head turning in Loetscher et al., 2008) and number processing in a standard SNARC design (Stoianov et al., 2008) can be explained via the working memory account: the long-term canonical number set is laid out in working memory along a spatial template and thereby becomes sensitive to exogenous spatial cues and/or attentional shifting (via for example) head turning.

5 We thank an anonymous reviewer for this suggestion.
